# Long-lasting virtual motorcycle-riding trainer effectiveness

**DOI:** 10.3389/fpsyg.2015.01653

**Published:** 2015-10-29

**Authors:** Giulio Vidotto, Mariaelena Tagliabue, Michael D. Tira

**Affiliations:** Department of General Psychology, University of PaduaPadua, Italy

**Keywords:** hazard perception, riding trainer, young novice riders, long-lasting learning, longitudinal study

## Abstract

This work aimed to test the long-lasting effects of learning acquired with a virtual motorcycle-riding trainer as a tool to improve hazard perception. During the simulation, the rider can interact with other road actors and experience the most common potential accident situations in order to learn to modify his or her behavior to anticipate hazards and avoid crashes. We compared performance to the riding simulator of the two groups of participants: the experimental group, which was trained with the same simulator one year prior, and the control group that had not received any type of training with a riding or driving simulator. All of the participants had ridden a moped in the previous 12 months. The experimental group showed greater abilities to avoid accidents and recognize hazards in comparison to their performance observed a year before, whereas the performance of the control group was similar to that of the experimental group 1 year before in the first two sessions, and even better in the third. We interpreted this latter result as a consequence of their prior on-road experience. Also, the fact that the performance of the experimental group at the beginning of the follow-up is better than that recorded at the end of the training—1 year before—is in line with the idea of a transfer from the on-road experience to the simulator. The present data confirm our main expectation that the effectiveness of the riding training simulator on the ability to cope with potentially dangerous situations persists over time and provides additional evidence in favor of the idea that simulators may be considered useful tools for training the ability to detect and react to hazards, leading to an improvement of this higher-order cognitive skill that persists over time. Implications for the reciprocal influence of the training with the simulator and the on-the road experience are discussed as well.

## Introduction

Motorcyclists are some of the most vulnerable road users. Crashes involving a motorcycle and at least one other vehicle account for over half of all motorcyclist deaths in the United States (National Highway Traffic Safety Administration, 2008). Motorcycle accident statistics show that in Europe and Australia, motorcyclists are more likely than any other vehicle users to be involved in collisions with a fixed object (European Transport Safety Council, 2007; Australian Transport Safety Bureau, 2008). Considering that the death rate of motorcycle riders increased from 2007 to 2011 in Europe (World Health Organization [WHO], 2013), in recent decades, a great deal of effort has been devoted to investigating the role that riding training programs play in reducing crash rates (Baldi et al., 2005).

Although several studies have indicated that hazard perception training in novice drivers leads to improved performance on hazard perception tests, it is still debatable whether or not such training will, in the long run, actually result in safer driving behavior and in fewer crashes. Research examining the effectiveness of motorcycle-training programs has generally yielded controversial results (Savolainen and Mannering, 2007). This is probably because, traditionally, rider-training programs have been based on teaching vehicle control skills (Chesham et al., 1993), rather than on the improvement of hazard perception skills, and no standard methods for evaluation exist (Daniello et al., 2009).

In a recent review, Savolainen and Mannering (2007) found an increase in fatalities among individuals whom had attended motorcycle safety classes. The authors hypothesized that there were several explanations for this result, which ranged from the use of ineffective course material to a decline in risk perception as a consequence of following the course or, furthermore, to the fact that riders attending the training were inherently less skilled than those who did not. Thus, on the basis of this study it remains unclear whether riding-skills training reduces the incidence rate of motorcycle crashes. On the contrary, McDavid et al. (1989)’s study supported evidence that trained riders tend to have fewer or less severe motorcycle accidents.

Other studies, which focused on driver education programs that trained the ability to detect areas of scenario from which hidden risks could emerge, demonstrated that these kinds of procedures are effective in improving scanning behaviors during on-road driving (Pradhan et al., 2006a) and suggested that this improvement will reduce the likelihood of a crash (Fisher et al., 2006).

Research in hazard perception is largely focused on young, novice, and inexperienced drivers; it was established that their hazard perception was poorer than that of their experienced counterparts. Chapman and Underwood (1998) found differences between experienced and novice drivers on hazard perception. On the basis of these differences, experienced and novice riders should also differ on riding behavior in response to hazards. Recently, Liu et al. (2009) confirmed this prediction by using an interactive motorcycle simulator. The authors found that experienced riders (relative to inexperienced or novice riders) crashed less often, were given better evaluations, and approached hazards at more appropriate speeds.

An increasingly popular approach is to explore the effectiveness of simulation-based training interventions on novice drivers by using driver simulators (Pradhan et al., 2006b; Wang et al., 2010; Morgan et al., 2011). Fildes et al. (1997) demonstrated that performance in driving simulators, as well as on-road driving performance, both correlate with respect to speed control and lateral placement. Recently, a study on the detection of driving hazards suggested that training in hazard perception could reliably transfer to real driving situations (Fisher et al., 2006). Whereas the abovementioned studies focus on car simulators, there is little evidence on the effectiveness of simulator-based motorcycle riding training (Liu et al., 2009; Hosking et al., 2010). Nevertheless, because hazard awareness and risk perception appear to be more critical for motorcycle riders than for car drivers (Haworth and Mulvihill, 2006), the main target of motorcycle training should be on the recognition of accident configurations rather than on avoidance maneuvers to use just before a collision (Hancock et al., 1990). Riding simulators seem to be particularly useful in this respect because they allow participants to be exposed to virtual dangerous situations without real risks. Indeed, Goode et al. (2013), in a recent review, concluded that evidence in favor of simulator efficacy is related to the training of higher-order cognitive skills, such as hazard perception.

In Italy, traditional training programs to obtain a motorcycle license to ride mopeds include theoretical lessons about traffic rules and, sometimes (but not always), a certain amount of on-road practice in the company of an expert trainer. In the first case, a novice licensed rider could, in principle, begin to go on-road with no experience at all. In the second case, the novice rider might encounter, during his/her training, some risky situations, but, obviously, the trainer, whom is able to communicate with him/her via headphones, had to prevent him/her from incurring a crash. So, first of all, the trainer had to intervene just before the hazard became a real possibility, and this might prevent the complete elaboration of the elements of the scene that should, in the following on-road experience, be recognized autonomously by the novice rider. Second, in the on-road practice, the majority of typical hazard scenarios are never encountered by the novices before getting their license, and this represents a serious element of inexperience that, in turn, is considered one of the human factors that concurs in determining the risk of crashes (Lin and Kraus, 2009). This is why efforts devoted to demonstrating the efficacy of other training modalities of riding training that allow familiarization with as many typical risky situations as possible through riding simulators (Isler et al., 2011; Vidotto et al., 2011) are considered extremely important.

When going into detail about the processes implied in safety riding/driving, agreement has been reached that one of the crucial underlying processes is the ability to anticipate potential hazards—i.e., to recognize the risk in advance—so as to be able to react promptly and prevent the accident (Deery, 1999; Rosenbloom et al., 2011). Consequently, one way to improve road safety education programs is to introduce training aimed at enhancing hazard perception. The most common way to do that is by showing video clips of real traffic scenarios and asking participants to detect hazards (Isler et al., 2011; Crundall et al., 2013). Employing this technique, Kinnear et al. (2013) demonstrated that, while watching a hazard scene, an anticipatory skin conductance response (SCR) can be recorded and that experienced drivers show more SCRs than novice drivers (i.e., whom have driven less than 1000 miles). Since SCR is considered a psycho-physiological response that is related to risk appraisal, and since the ability to recognize risks in advance is, very often, the only chance to avoid accidents, their results seem to demonstrate that passive experience—just watching a video clip—enhances the probability to avoid accidents when hazards are developing (Kinnear et al., 2013).

Simulators represent one possible additional tool (among others) for road safety education programs and their effectiveness has been debated in the last decade. Goode et al. (2013) carried out an overview of studies focused on virtual simulators in a variety of applications (from the training to drive tanks to the training of teamwork skills in emergency situations) and concluded in favor of the effectiveness of simulators in higher-order cognitive skills training. Indeed, they reported studies focused on hazard perception (one of these higher-order cognitive skills), showing that participants trained with simulators seem able to perceive and respond to hazards more appropriately than controls. Meuleners and Fraser (2015), by focusing on the relative validity of a driving simulator, demonstrated the coherence between on- and off-road behaviors with reference to some driving variables. Finally, Shechtman et al. (2009) approached the problem of the driver response validity by comparing errors made by the same group of participants— both on road and with a simulator—at a street intersection and provided evidence in favor of the relative validity of the simulator.

On the basis of these considerations, Vidotto et al. (2011) carried out a study to investigate the effect of a riding simulator on teenagers—that is to say, novice riders—before they took the riding license exam. They demonstrated the effectiveness of this tool in enhancing hazard avoidance due to an improvement in the ability to identify potentially dangerous situations in a virtual environment.

On the other hand, another important contribution with which to understand the implication of the use of a simulator in riding education programs would be to directly investigate the risk of the accident rate in riders trained with simulators in comparison to riders trained with other road safety education programs in a wide time window. To reach this goal, one prerequisite is to assess the duration in time of the effects of the trainings provided through simulators. If the effect of simulators decreases over time, it should be unnecessarily onerous to carry out longitudinal studies on large samples to calculate accident rates.

The aim of the present research was to assess the long-term persistence of the outcomes of the training shown in Vidotto et al. (2011). In other words, we wondered whether the learning effects observed in young novice riders trained through the Honda Riding Trainer (HRT) in the previous study (training phase) persisted 1 year after (at the follow-up—the present research), comparing the performance of a subgroup of the original research at which we re-administered the HRT procedure (our experimental group) with those of a control group of equivalent participants (in term of gender, age, and riding experience) who had only received traditional theoretical training for riding license achievement 1 year before. Our hypothesis was that when the training effect is long lasting, participants in the experimental group should show, in the very first sessions of the follow up, an equivalent or better performance than that recorded at the end of the first training phase; this improvement should indicate that the previous learning consolidates over time. Conversely, in the case of an overall decay of learning effects in the follow-up, we expected a learning curve resembling that recorded 1 year before. Thus, we expected that the experimental group maintained at least the higher level of HRT performance (reached in third session of the training phase) 1 year later at the follow-up. We also expected that the control group would show, at the follow-up, a trend of performance comparable with that of the experimental group in the training phase, but worse than that of the experimental group at the follow-up.

## Materials and Methods

### Participants

Forty-eight participants took part in this study: 24 (6 females and 18 males, 15–16 years old) as the experimental group and 24 (6 females and 18 males, 15–16 years old) as the control group. Motivation to take part in the project was supported by a reward, which was the opportunity to earn school credits that the participants could use in their final examination.

We randomly selected the participants for the experimental group among the students of two schools who agreed to take part in the follow-up (i.e., the present research) of a simulator riding training program that was previously completed while they were studying to obtain their riding license [the training phase; i.e., the original study of Vidotto et al. (2011)].

The 24 participants of the control group were randomly selected among the students of the same schools who had not participated in the study of Vidotto et al. (2011), but who had attended the same traditional educational program in order to obtain a moped license. Selection was performed in order to match the participants’ characteristics of the experimental group for gender and age.

All of the participants (both groups) were naïve about the aim of the research and had normal or corrected-to-normal vision. All of them had obtained the moped riding license and driven on the road during the year preceding the present follow-up study. No other inclusion criteria were considered.

### The Riding Simulator

The HRT is a motorcycle simulator powered by PC Pentium 4-based computer equipment connected to a 19-inch LCD monitor at 1024 × 768 resolution and motorcycle controls (i.e., a handlebar and foot pedals). The screen was located in front of the rider at a distance of approximately 80 cm; the horizontal angle of the visual field was 27.2°, and the vertical angle was 21.7°.

The simulator technology was created as a part of an integrated motorcycle safety concept that focused on addressing the human factor element. The HRT was developed to provide the rider with better awareness of traffic situations and the ability to build up skills in defensive riding, a riding style based on the anticipation of other road users’ behavior. Moreover, it proved to be a useful tool in research to investigate a variety of cognitive aspects related to motorcycle-riding skills (Di Stasi et al., 2009; Liu et al., 2009; Armartpundit et al., 2010; Symmons and Mulvihill, 2011).

The HRT offers a wide range of scenarios within urban, seaside, mountain, and rural areas in 16 different courses: two training courses (that are used to become accustomed to the HRT; in these courses, no other vehicles are present on the road), six courses on main streets, five courses on secondary streets, and five courses for “touring” (city, highway, seaside, mountain, and neighborhood). Before each ride, several characteristics are set up: engine size (small, medium, and large), transmission type (manual or automatic), setting (main street, secondary street, and rural environment), and light conditions (day, night, or fog).

There are eight hazard scenes in each course, except for one that has only seven scenes. They reproduce the traffic configurations according to a study that analyzed 921 motorcycle accidents over the course of 3 years to identify the most common motorcycle collision scenes (MAIDS, 2004). Hazards include situations in which the trainee has to go from a parking area onto the roadway; paying attention in the mirror to the vehicle approaching from behind; points in which the trainee is starting to turn left while a preceding vehicle is also turning left, too, which can prevent the trainee from seeing another motorcyclist who is occupying the intersection; or situations in which the trainee has to turn to the left from a narrow road onto a two-way street while a truck obstructs his view. Other scenarios recreate situations that allow individuals to experience danger from a sudden change in pedestrians’ behavior, such as a child who begins crossing the road but, realizing that the traffic light is changing to yellow, starts to come back onto the crosswalk. Another scenario involves children running alongside the sidewalk who suddenly change their trajectory while crossing the road, or again, along a residential road, a child appears suddenly from behind a wall while chasing a ball. Moreover, situations in which a parked vehicle creates a hazard by suddenly starting or opening the door are also depicted.

The HRT software has an algorithm that can refer to the motorcycle operating speed. In this way, the generation of dangerous scenes is timely, even if each rider’s speed is different. Hazards are set along the course at specific moments, and the participant is guided on the predetermined course via voice announcements. There can be deviations and/or accidents, but the rider always arrives at the end of the predetermined course. Whenever an accident occurs, the software automatically replays the events that led up to the crash. Each course ends with a replay of the entire course that integrates comments and advice on riding behavior and a final spreadsheet summarizing what has already been explained during the replay.

### Procedure

As explained before, we will refer to the data collected for the experimental group in the study of Vidotto et al. (2011) as to the training phase, and to the data collected in the present research as to the follow-up. In order to compare the performance of the experimental group in the training phase on HRT with that recorded 1 year later for the same group and for the control group, the participants (in both groups) completed three sessions of four courses in three different weeks using the same procedure as in Vidotto et al. (2011). Each session included four courses, for a total of 12: six courses on main streets, five on secondary streets, and one in neighborhoods. Considering that there were eight hazard scenes in each course, except for one that had only seven, participants had to cope with a total of 95 hazard scenes.

As depicted in **Table 1**, there was a 1-week span of time between the first and the second sessions and 1 week between the second and the third session. The courses’ presentation order was randomized using a Latin square-like design so that each course was included in each order position the same number of times (i.e., each course was presented with the same frequency in all of the order positions). This method ensured both the estimation of the practice effect and the control of the stimuli sequence effect (Miceli et al., 2008). All participants had a setup with small engine size, automatic transmission, and daylight conditions, and they always began the first session with the two training courses in which no other vehicles were present on the road (preliminary training). The completion of all 12 courses took approximately 1 hour, and each participant was tested on an individual basis. An instructor, who was always present during the training, supervised all participants. Although he was allowed to speak to the participants during the training, the interaction was kept neutral and as limited as possible. At the beginning of the first session, the instructor provided technical information. During the courses he could answer questions, but he was not permitted to remind the participants about the content of the voice announcement. The instructor was allowed to intervene whenever a participant demonstrated a reckless attitude, and he was asked to take notes on riding behavior.

**Table 1:** The experimental design including the training phase occurred 1 year before the present research.

	Training phase			Interval	Follow-up		
	First week	Second week	Third week	1-year	First week	Second week	Third week
Experimental group	Theoretical Lessons			Riding moped			
	Virtual training session-1	Virtual training session-2	Virtual training session-3		Virtual training session-4	Virtual training session-5	Virtual training session-6
Control group	Theoretical lessons			Riding moped	Virtual training session-1	Virtual training session-2	Virtual training session-3

In addition, participants had to fill out a 5-item questionnaire in which they were asked about their riding habits in the last 12 months, such as (1) “What is your mean speed when you ride on an urban street?;” (2) “What is your mean speed when you ride on an extra-urban street?;” (3) “What do you think about your riding style?;” (4) “How much safe do you think your riding is?;” and (5) “How much of an expert do you think you need to be when riding?” Finally, participants were asked how many kilometers they used to ride per week.

The research was conducted in conformity with the ethical standards of the field and was approved by the school headmaster in accordance with the relevant regulatory standards of the educational purposes and by the school supervisor for the road safety education program that also included the theoretical lessons administered by local police officers for moped license achievement. Formal consent to take part in the study was obtained from participants and their parents.

### Generalized Linear Mixed Models

Analyzing crash-frequency data involves several methodological issues and statistical models (Lord and Mannering, 2010; Savolainen et al., 2011). Let us consider a short overview of a few basic aspects of some useful models to analyze count data, such as crash frequency.

Generalized linear models (GLMs) are an extension of the linear models; more specifically, they are a class of fixed-effects regression models for different types of dependent variables (such as continuous, dichotomous, counts, etc.) so that they include not only linear but also logistic Poisson and several other kinds of regression models (Nelder and Wedderburn, 1972). It is worth noting that, by using fixed effects in linear models, we assume that all observations are independent of each other; unfortunately, these models are not appropriate for the analysis of several types of correlated data structures, such as longitudinal data. In longitudinal designs, we could have repeated observations nested within subjects. To analyze such data, subject effects can be added into the regression model to account for the correlation of the data. The resulting model is a mixed model that includes the usual fixed effects for the regressors plus the random effects (Laird and Ware, 1982).

Mixed models for continuous normal outcomes have been extensively studied and applied for non-normal data as well; many of these developments fall under the title of generalized linear mixed models (GLMMs), which extend GLMs by the inclusion of random effects in the predictor (Engel and Keen, 1994). A random-intercept model, which is the simplest mixed model, augments the linear predictor with a single random effect for each single subject. These random effects represent the influence of each subject on the repeated observations that is not captured by the observed covariates. Various methods for incorporating and estimating the influence of the random effects have been recently considered and implemented in R-language (Pinheiro and Bates, 2000; Bates et al., 2012). For all of these reasons, mixed logit models are a valuable alternative to other standard commonly used models to analyze crash-frequency data.

## Results

As previously illustrated, the participants involved in our experiment had to ride in a virtual environment with 12 different courses. Each course had either seven or eight different hazard scenes in which the participants had to avoid causing an accident. Binomial response data were considered with the count of the avoided hazards out of the total number of hazard scenes. Moreover, 12 repeated measures for each subject, referring to the 12 different courses, had to be contemplated. Given that the focus of the research was on learning, we were interested in the changes due to order presentation, but we also had to balance response data introducing in the model the information about the difficulty of the different courses. As a final model, we had the avoided hazards as a dependent variable, whereas the phases or sessions over time were a within-subject factor. An index related to the difficulties of the courses was also introduced as a covariate. This index was based upon the average proportion of accidents for all of the hazard scenes occurring in a course (i.e., the ratio of the count of the accidents to the total number of hazard scenes) observed in a sample of more than 400 previously tested individuals.

Statistical analyses were conducted by using R Core Team (2011) and, as previously explained, logistic GLMMs were chosen for the analyses because they deal with non-normal responses (i.e., binomial) and repeated measures (i.e., phases/sessions over time). It is worth noting that because of the dichotomous nature of the dependent variable and the constant number of hazard scenes; inferential results coincide when based on the number of accidents or on the number of avoided hazards. One participant of the experimental group (16) was excluded from the following analyses for an artifact in processing the HRT data during his first three sessions.

**FIGURE 1:**
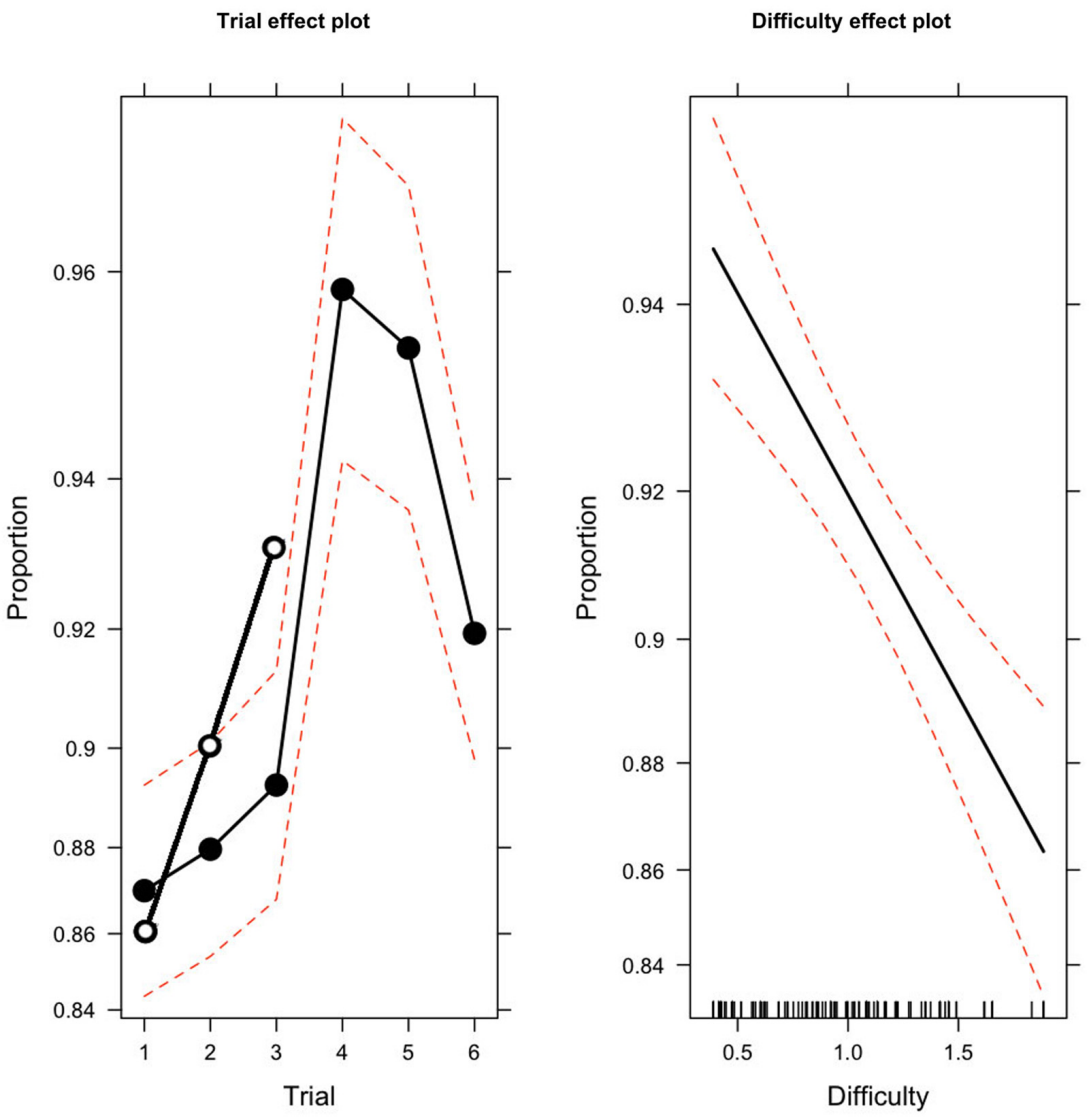
**Plot on the **Left**: proportion of avoided hazards (*y*-axis) during the six sessions (*x*-axis) for the experimental group (solid line with black circles) and the three sessions for the control group (solid line with white circles).** For the experimental group, Trials 1 to 3 refer to the three sessions of the training phase and 4 to 6 to the three sessions of the follow-up. For the control group, Trials 1 to 3 refer to the three sessions of its first and unique exposure at the Honda Riding Trainer (HRT) procedure during the follow-up. Plot on the **Right**: predicted proportion of avoided hazards (*y*-axis) against the index of difficulty of the courses (*x*-axis). Ninety-five percent point-wise confidence bands are shown as broken red lines.

Concerning the experimental group, **Figure 1** displays, on the left side, the mean proportion of the avoided hazards (a solid line with black circles) for the 3 sessions (trials 1–3) during the training phase (0.87, 0.88, 0.89^1^) and the three sessions (trials 4–6) at the follow-up (0.96, 0.95, 0.92). The greatest amount of improvement occurred between the last session of the training phase and the first session at the follow-up (0.07). **Figure 1** also displays, on the right side, the linear link between the mean proportions of avoided hazards and the difficulty index of the 95 hazard scenes. Dashed lines always represent the 0.95 confidence intervals.

The results of the logistic GLMM analysis carried out on data of the experimental group showed how the means increased from the first to last session [χ^2^(5) = 67.80; *p* < 0.001], following a trend with strong linear (*z* = 6.54; *p* < 0.001), quadratic (*z* = -3,85; *p* < 0.001), and cubic (*z* = -4.62; *p* < 0.001) components. The results also showed the significant effect of the covariate [χ^2^(1) = 25.28; *p* < 0.001], denoting that the difficulty level of the completed course affects the observed performance (**Figure 1**, right side). It is worth noting that the mean proportion of the avoided hazards in the follow-up was considerably higher than during the training phase [0.88 vs. 0.94; χ^2^(1) = 7.64; *p* < 0.001] with a difference of 0.06. No other statistically significant effects were observed.

On the left side of **Figure 1**, the mean proportion of the avoided hazards of the control group (a solid line with white circles) for the three sessions (trials 1–3) during the follow-up (0.86, 0.90, 0.93), is displayed as well. The results of the logistic GLMM analysis showed how means increased from the first to the third session [χ^2^(2) = 22.99; *p* < 0.001] following a trend with a strong linear (*z* = 4.63; *p* < 0.001) component. Also, for the control group, the results showed the significant effect of the covariate [χ^2^(1) = 22.40; *p* < 0.001], denoting that the difficulty level of the completed course affects the observed performance.

Comparing the results of the two groups in the first three sessions, we observed that there was no significant difference between the two groups at the first and second session (respectively, *z* = 0.53; ns; *z* = –0.33; ns), while the control group showed a higher proportion of avoided hazards during session three (*z* = –2.86; *p* < 0.005).

Comparing the results of the control group with the ones of the experimental group at the follow-up, we observed that the control group showed a lower proportion of avoided hazards at the first and second session (respectively, *z* = 6.51; *p* < 0.001; *z* = 3.86; *p* < 0.001), while there was no significant difference during session 3 (*z* = –0.99; ns).

Concerning information about the amount of on-road experience, for some technical coding problem we were able to retrieve only those of the experimental group; of the 24 participants, we discarded three questionnaires due to missing data. As to the remaining 21 participants, two participants used to ride 1–10 km per week, three participants rode 11–30 km per week, 11 rode 31–50 km per week, four participants rode 51–100 km per week, and the last participant declared to ride more than 100 km per week.

Thus, to check whether the overall performance of the participants belonging to the experimental group was related to the amount of exposure or the frequency of moped use in the past 12 months, we carried out a logistic GLMM analysis on the avoided hazards with amount of exposure as the three levels of the between-subjects factor (less than 30 km per week, 31–50 km per week, and more than 50 km per week). The mean proportion of avoided hazards for the three groups of participants was, respectively, 0.94, 0.94, and 0.96. The amount of exposure factor did not reach significance.

Finally, we compared the two groups on information about the riding habits and we found no statistically significant differences between the experimental group and the control group on these measures, except for the item “How much safe do you think your riding is?,” in which the difference between 3.8 (experimental group mean) and 4.24 (control group mean) was significant with *p* < 0.001. This could be in line with the idea that the participants of the experimental group have developed a higher hazard perception and overall hazard awareness due to the previous exposure to the simulator; that is, just the effect one would like to obtain in road safety training so as to improve, in turn, the ability to perform more adequate and defensive riding behaviors. No differences in the other measures were found, confirming that the experimental group and the control group have similar driving habits.

## Discussion

The present study assessed the hypothesis of persistence over time of the learning effect acquired with the HRT simulator. Three crucial comparisons were used to test this hypothesis: The comparison between HRT performance of the experimental group in the three sessions of the training phase (1 year before with no riding experience) and the HRT performance of the same group at the follow-up 1 year later (with 1 year of experience riding on the road); the comparison between the HRT performance of the control group at the follow-up (its first and unique experience with the simulator, but with one year of riding-on-the-road experience) with that of the experimental group at the training phase 1 year previously (its first experience with the simulator); the comparison between HRT performance of both groups at the follow-up (both groups with 1 year of riding-on-the-road experience).

The first comparison provides direct evidence about the persistence in time of the learning acquired with the simulator by the experimental group. The results just described seem to be consistent with the hypothesis of the long-term persistence of the outcome of the training in that the experimental group not only maintains the higher performance reached at the end of training session soon at the beginning of the follow-up (as we expected) but, it also reveals even better performance in the first session of the follow-up than in the last session of the training.

Note that, in the case of non-specific contextual effects of familiarization with the device, the improvement observed at the beginning of the follow-up should not be higher than the improvement recorded at the end of the training phase 1 year prior. On the contrary, the results showed that the greater amount of improvement in riding performance occurs between the last session of the training phase and the first session of the follow-up. Indeed, it might be argued that the simplest and most parsimonious explanation is that the improved riding performance is a direct result of the 12 months of additional moped riding experience.

In addition, the comparison between the performance of both groups at their first experience with the simulator clearly showed that the control group behavior resembled that of the experimental group in the first two sessions, but it is better in the third session. Also in this case, since the groups were equivalent with reference to virtual experience but different with respect to on-road experience, it seems that on-road experience affects HRT performance.

Moreover, when we compared the HRT performance of the control group with the last three sessions (the follow-up) of the experimental group, thus matching the effect of the on-the-road experience, the participants with no prior training with the simulator showed a significantly lower performance in two of the three sessions considered.

Thus, data seem to indicate not only that whatever was learned is still active one year later, but also that the effect of learning was enhanced over time. The only way to explain such a result is by means of the concept of consolidation, which is a process during which new knowledge is gradually incorporated into internal representations, and then reactivated when some elements of specific experiences appear anew so as to be used to govern related behaviors (McClelland et al., 1995).

This strengthening or consolidation effect over time has been shown in several studies focused on learning effects, no matter the cognitive process implied. For instance, Tagliabue et al. (2000) demonstrated the existence of consolidation in the effects of learning with spatial compatibility tasks (in which the spatial position is the relevant dimension for the response selection) on a Simon task in which spatial position modulates the response despite its irrelevance in the task. The result of the consolidation of learning – demonstrated also in cross-modal experimental conditions (Tagliabue et al., 2002) - is interpreted as an index of cognitive plasticity. It is worth noting that plasticity shown in the aforementioned study referred to spatial information; indeed, it is evident that spatial learning plays a crucial role in riding training as well.

It can be pointed out that the participants of the experimental group of the present study continued to train themselves in their daily lives after the end of the training phase with the simulator, and this might be the cause of the consolidation of the learning effect recorded, independently of the frequency/intensity of moped use (since the analysis on the overall performance of the participants did not show evidence of a relation between the amount of moped riding experience and the participants’ performance in the follow-up). In this circumstance, it would be as to whether the participants had done a “rehearsal” of the simulator experience while riding with their real motorcycle during the year, so as to show additional improvement in performance once the simulator sessions were re-administered one year later. If this were the case, it would indicate a generalization process backward from real life to the simulator performance, which is impossible if a previous generalization, from either virtual experience to real life, did not occur.

Even more, when compared with the experimental group at its first experience during the training phase, the participants of the control group show higher improvement at the third session. Thus, it is as if the experience acquired in the real-world environment had played a certain role after the initial sessions mainly related to becoming acquainted with the virtual environment. In other words, the control group may have earned a higher score than the experimental group at the third session because of its past on-road riding experience.

Conversely, comparing the control group sessions with the experimental group sessions at the follow-up, we observe an opposite result for the experimental group: the improvement in the experimental group performance is more evident at the beginning of the follow-up (that is, just after their exposure to the real-world environment). This improvement is diminished at session two, and it disappears at session three.

Taken together, the results of the better performance of the control group in its third session and of the better performance of the experimental group in the first two sessions of the follow-up are again coherent with the hypothesis that the improved riding performance is related to the 12 months of riding experience.

Finally, even though the performance of the experimental group after 1 year is overall the best one, and it could be interpreted as evidence of the fact that introducing the training session prior to the on-road practice is advantageous, there is, on the other hand, a result that needs to be explained: The experimental group did not maintain that score along the three sessions at the follow-up. A short interview at the end of the follow-up suggests the idea that boredom and declining motivation played a major role, as the participants of the experimental group have had an overexposure to the HRT.

## Conclusion

High mortality and, in general, high accident rates in riding powered two-wheel vehicles have a significant impact all over the world. The consequences of riding crashes are very often more serious than those following driving accidents because of the greater vulnerability of motorcyclists. This is the reason why studies on riding abilities have attracted the attention of different types of specialists, from those who design safety devices for vehicles to cognitive psychologists who devote a significant amount of energy to investigate all of the cognitive abilities implied in riding (Lin and Kraus, 2009) and to identify best practices for road safety education to maximize riding efficacy to prevent accidents (Baldi et al., 2005).

The entire project moved on from this aim and started from the idea that simulator training provides the opportunity to cope with unforeseen and dangerous road events that would be unsafe to experience in a real-life environment. This opportunity, in turn, has been demonstrated to improve the ability of novice riders to recognize hazard situations and to react in such a way as to avoid risks (Vidotto et al., 2008, 2011). Indeed, risk appraisal and recognition represent the most important underlying processes for road safety driving or riding (Deery, 1999; Rosenbloom et al., 2011; Vidotto et al., 2011; Crundall et al., 2013). This ability has been also demonstrated to be related to psycho-physiological responses, such as SCRs, that seem to be enhanced with experience (Kinnear et al., 2013). Thus, allowing inexperienced drivers to gain experience in safe conditions should improve their ability to avoid accidents on the road.

Surely, the use of virtual simulators in educational road safety programs is just one of some other possibilities, but any effort aimed at understanding the validity and efficacy of the learning effects that this tool may provide could represent a chance to reduce road accident rates that, among two-wheeled vehicle users, often lead to more serious outcomes.

However, besides demonstrating that virtual training is highly effective, it was crucial to prove that learned abilities acquired in such a way are retained over a longer period of time. The present results show that the virtual riding training program provides a long-lasting learning effect and offers some evidence that experience on the road transfers to simulator performance since the main result of the present study is the enhancement of learning effects as measured by means of the simulator during the year between the training and follow-up phases. The consolidated effect recorded at the follow up, especially in the hypothesis that it is due to the on-road experience, constitutes additional evidence of the transfer of learning among virtual and real experiences.

In conclusion, the present research provides evidence that the HRT simulator could be considered a useful tool for riding training in that it allows novice riders to cope with potentially dangerous road situations, inducing long-lasting improvement in the ability to recognize in advance hazards so as to avoid risky behaviors. Thus, the next step in order to arrive at a complete understanding of the implication of the use of simulators in road safety training—that is, the assessment of the rate of crash/accident risk probability on the road of people trained with virtual road simulators vs. other training modalities—remains a challenge for future research.

## Conflict of Interest Statement

The authors declare that the research was conducted in the absence of any commercial or financial relationships that could be construed as a potential conflict of interest.

## Footnotes

1 Note that these data are comparable with those of the entire experimental group of the original study of Vidotto et al. (2011), in which the mean proportions of avoided hazards for the three sessions during the training phase were 0.83, 0.88, 0.91, respectively.
